# Supplementary material for: Probing into the In-Situ Exsolution Mechanism of Metal Nanoparticles from Doped Ceria Host
Source: Nanomaterials (Basel). 2021 Aug 19;11(8):2114. doi: 10.3390/nano11082114 (PMC8398560; doi:10.3390/nano11082114)
Supplement: Supplementary file 1 [file nanomaterials-11-02114-s001.zip › nanomaterials-1334405-supplementary.pdf]

Supplementary Materials

Probing into the In-Situ Exsolution Mechanism of Metal Nanoparticles from Doped Ceria Host

Lifang Zhang [1], Weiwei Ji [1], Qiyang Guo [1], Yu Cheng [1,*], Xiaojuan Liu [2,3], Hongbin Lu [1,*] and Hong Dai [1,*]

[1] School of Chemistry and Chemical Engineering, Nantong University, Nantong 226009, China; lfzhang@ntu.edu.cn (L.Z.); 1808041069@stmail.ntu.edu.cn (W.J.); gyguo@ntu.edu.cn (Q.G.)

[2] State Key Laboratory of Rare Earth Resource Utilization, Changchun Institute of Applied Chemistry, Chinese Academy of Sciences, Changchun 130022, China; lxjuan@caic.ac.cn

[3] University of Science and Technology of China, Hefei 230026, China

* Correspondence: chengyu@ntu.edu.cn (Y.C.); luhb@nju.edu.cn (H.L.); dh123@ntu.edu.cn (H.D.)

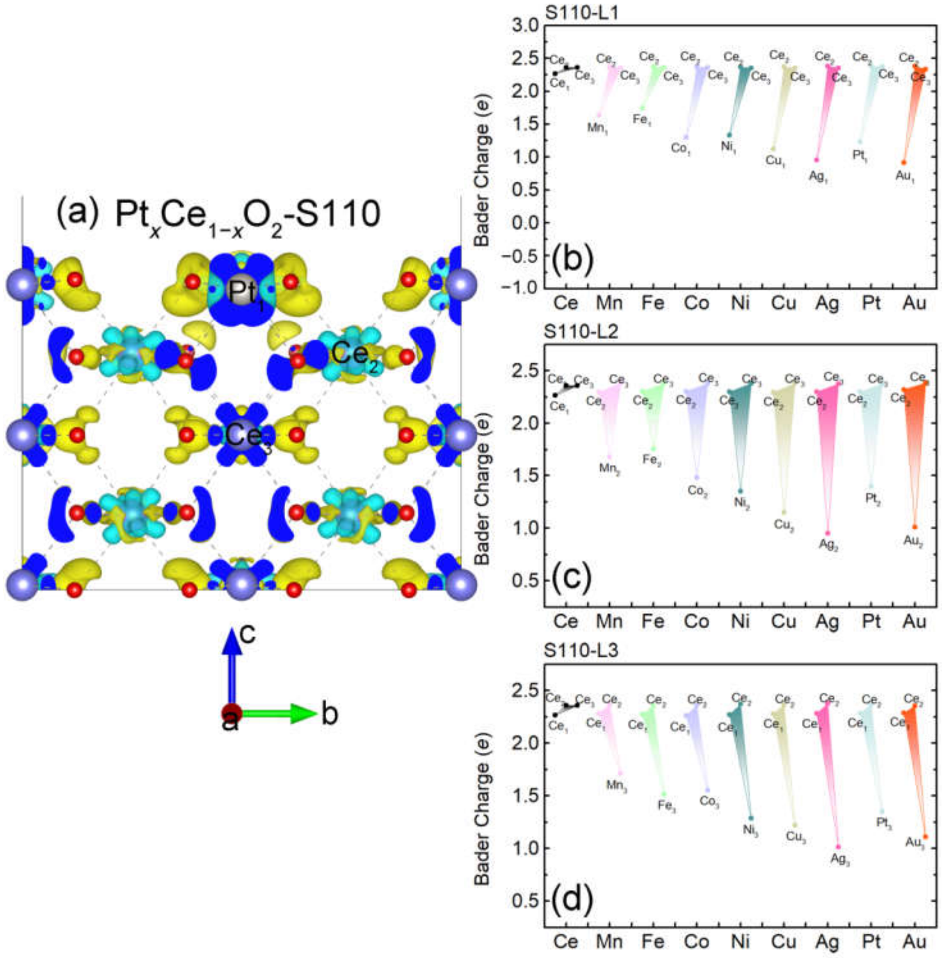

Figure S1. (**a**) The 3D-plot of charge density difference with an isosurface value of 0.02 *e*/Bohr^3 for $Pt_xCe_{1-x}O_2$-S110 with Pt at L1. (**b**–**d**) Evolution of Bader charge for $M_xCe_{1-x}O_2$-S110 with the dependence of Mn, Fe, Co, Ni, Cu, Ag, Pt and Au: (**b**) M in L1, (**c**) M in L2 and (**d**) M in L3.

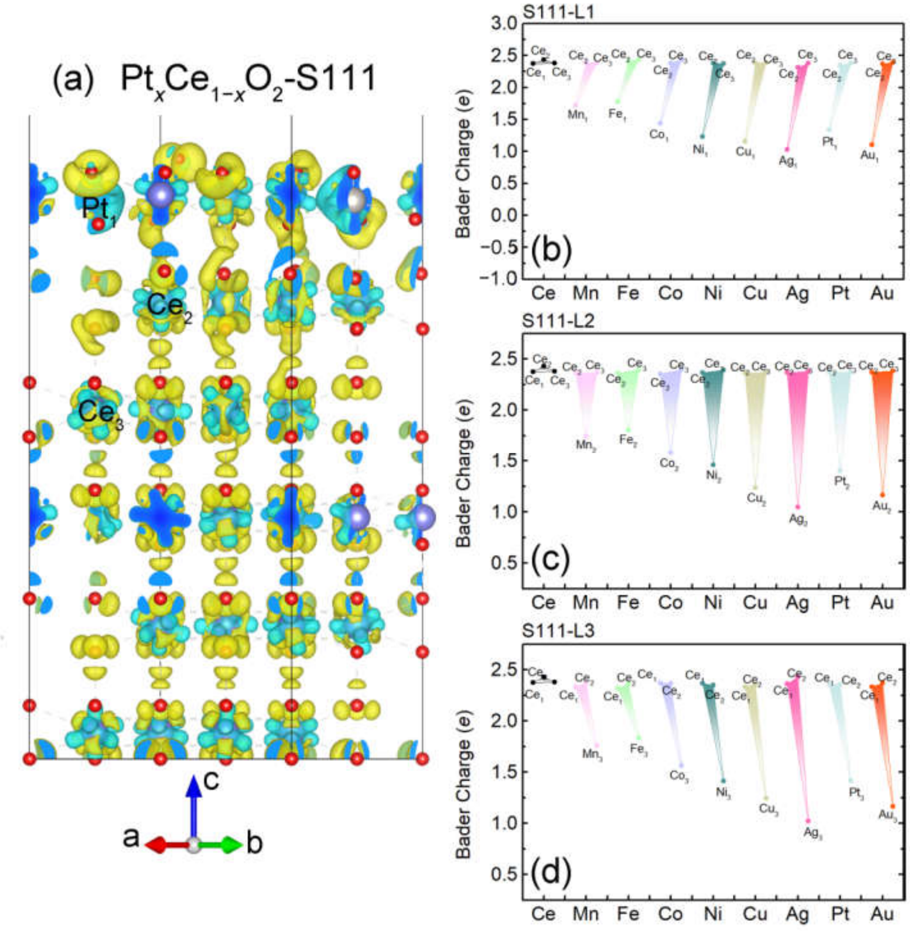

Figure S2. (**a**) The 3D-plot of charge density difference with an isosurface value of 0.02 *e*/Bohr^3 for Pt$_x$Ce$_{1-x}$O$_2$-S111 with Pt at L1. (**b–d**) Evolution of Bader charge for M$_x$Ce$_{1-x}$O$_2$-S111 with the dependence of Mn, Fe, Co, Ni, Cu, Ag, Pt and Au: (**b**) M in L1, (**c**) M in L2 and (**d**) M in L3.

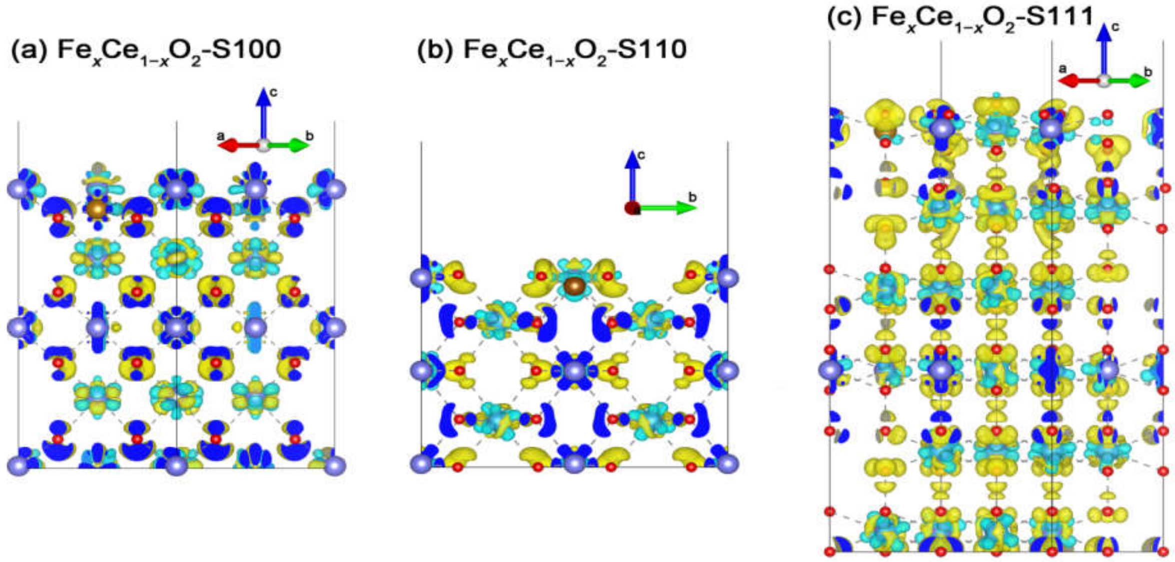

Figure S3. The 3D plots of charge density difference with an isosurface value of 0.02 *e*/Bohr^3 for Fe$_x$Ce$_{1-x}$O$_2$ with different oriented surfaces: (**a**) Fe$_x$Ce$_{1-x}$O$_2$-S100, (**b**) for Fe$_x$Ce$_{1-x}$O$_2$-S110 and (**c**) Fe$_x$Ce$_{1-x}$O$_2$-S111.

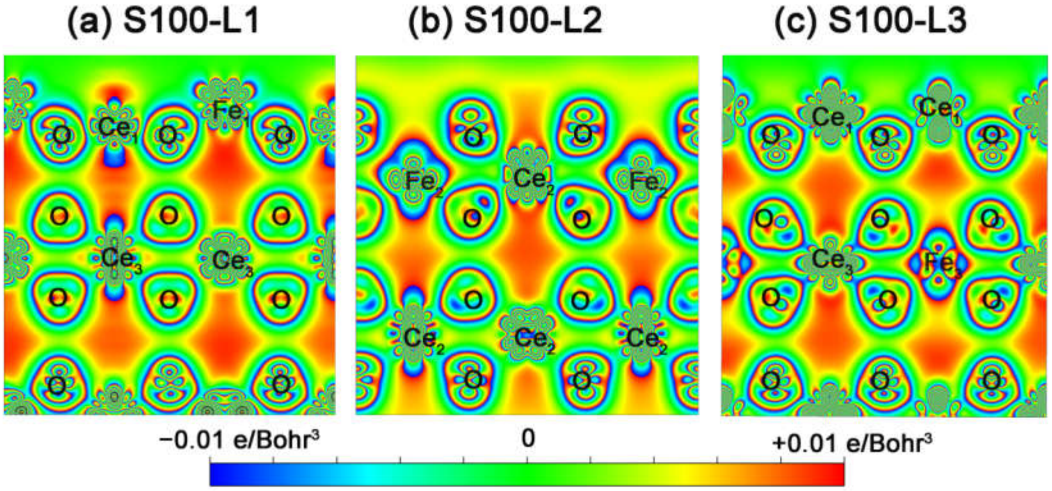

Figure S4. 2-D charge density difference plots along the (001)-direction ($\Delta n(\mathbf{r})$ in units of e/Bohr^3) for 1.5 × 1.5 × 1 $Fe_xCe_{1-x}O_2$-S100, which with Fe (**a**) on surface layer L1, (**b**) in second layer L2 and (**c**) in the 'bulk' layer L3.
